# Super Resolution Network Analysis Defines the Molecular Architecture of Caveolae and Caveolin-1 Scaffolds

**DOI:** 10.1038/s41598-018-27216-4

**Published:** 2018-06-13

**Authors:** Ismail M. Khater, Fanrui Meng, Timothy H. Wong, Ivan Robert Nabi, Ghassan Hamarneh

**Affiliations:** 1 Medical Image Analysis Lab, School of Computing Science, Simon Fraser University, Burnaby, BC V5A 1S6 Canada; 2 Department of Cellular and Physiological Sciences, Life Sciences Institute, University of British Columbia, Vancouver, BC V6T 1Z3 Canada

## Abstract

Quantitative approaches to analyze the large data sets generated by single molecule localization super-resolution microscopy (SMLM) are limited. We developed a computational pipeline and applied it to analyzing 3D point clouds of SMLM localizations (event lists) of the caveolar coat protein, caveolin-1 (Cav1), in prostate cancer cells differentially expressing CAVIN1 (also known as PTRF), that is also required for caveolae formation. High degree (strongly-interacting) points were removed by an iterative blink merging algorithm and Cav1 network properties were compared with randomly generated networks to retain a sub-network of geometric structures (or blobs). Machine-learning based classification extracted 28 quantitative features describing the size, shape, topology and network characteristics of ∼80,000 blobs. Unsupervised clustering identified small S1A scaffolds corresponding to SDS-resistant Cav1 oligomers, as yet undescribed larger hemi-spherical S2 scaffolds and, only in CAVIN1-expressing cells, spherical, hollow caveolae. Multi-threshold modularity analysis suggests that S1A scaffolds interact to form larger scaffolds and that S1A dimers group together, in the presence of CAVIN1, to form the caveolae coat.

## Introduction

Understanding the structure of macromolecular complexes is critical to understand the function of subcellular structures and organelles. X ray crystallography and nuclear magnetic resonance spectroscopy report on protein structure at the atomic level; recent technical advances in cryo-electron microscopy have enabled structural visualization of macromolecular biological complexes at near atomic resolution^1^. While fluorescence microscopy has been extensively used to study subcellular structures and organelles, its application to structural analysis of macromolecular complexes has been restricted by the diffraction limit of visible light (∼200–250 nm). Super-resolution microscopy has broken the diffraction barrier and, of the various super-resolution approaches, the best resolution is obtained using single molecule localization microscopy (SMLM). Based on the repeated activation (blinking) of small numbers of discrete fluorophores (using, for instance, PALM, dSTORM or GSDIM), whose precise localization is determined using a Gaussian fit of the point-spread function (PSF), SMLM provides ∼20 nm X-Y (lateral) resolution and, with the addition of an astigmatic cylindrical lens into the light path, ∼40–50 nm Z (axial) resolution^2,3^.

However, development of analytical tools to interpret the point distributions generated by SMLM is in its infancy^4^. Surface reconstruction and density plots of 3D super-resolution data assume idealistic, noise-free setting and lack quantification^5^. Ripley’s K, L, and H-functions and univariate/bivariate Getis and Franklin local point pattern analysis have been used to analyze super-resolution data for different applications^6–12^. While useful for global cluster analysis, these second-order statistics have limited ability to deal with localized shape and size properties of homogenous clusters. Moreover, calculating the Ripley’s function is computationally intensive making it impractical for handling millions of points^13^. It is also known that Ripley’s function underestimates the number of neighbors for points near the boundary of the 2D or 3D study area (known as the edge effect)^14^. Several correction methods were proposed to solve the edge effect problem but at the expense of even further increase in computational complexity making it unfeasible to scale to SMLM big-data.

Density-based methods (e.g. DBSCAN, OPTICS) and Bayesian approach combined with Ripley’s functions^15,16^ retain the inability to deal with varying cluster densities and sensitivity to prior settings and noisy events. DBSCAN has several parameters that must be carefully set and its runtime scales quadratically with the number of points (e.g. for SMLM data, DBSCAN can take several hours to run)^17^. Voronoi tessellation depends on Voronoi cell areas to segment clusters and has limited multiscale capability^13,18^. Griffié *et al*.^19,20^ proposed a parameter-free cluster analysis method that is based on Bayesian prior probabilities. Their method is sensitive to the prior settings and processing time to analyze one data set, 30 2D ROIs of size 3 × 3 *μm*^2^ each at low density, is ∼19 hours, and thus does not scale to large SMLM datasets^19^. Other methods focused on molecular counting by studying photoactivatable fluorescent proteins (FPs) and their blinking behavior, which is based on the photokinetic model of the FPs^21,22^. Moreover, Krüger *et al*.^22^ used the molecular count per cluster and cluster shape, size, and overlap to select a set of clusters from SMLM data for quantitative analysis. Nieuwenhuizen *et al*.^23^ proposed a method that measures the average number of localizations per fluorophore from spatial image correlation during acquisition.

The multiple blinking of a single fluorophore can cause an overcounting artifact that might bias the quantification of SMLM data and consequently lead to misinterpretations of the biological findings. Keller *et al*.^24^ summarize the methods used to mitigate the overcounting artifacts using temporal and spatial grouping techniques. The temporal methods require detailed information about the photokinetic rates and the dark time for the fluorophores used and not all the photoswitchable probes are photodetectable^21^. Annibale *et al*.^25^ uses photophysical information to combine events that are assumed to be originating from the same fluorophore. Sengupta *et al*.^26^ proposed a method that combines pair-correlation analysis with PALM to distinguish between spatial localizations from a single protein (i.e. nano-clusters) and clusters of proteins. Recently, a method adopted by two groups^27,28^ is based on determining the ratio of clustered area per image and the density of localizations per clustered area at various densities, either by changing the labeling density^27^ or using temporal accumulation analysis of the acquired localizations (blinks)^28^.

We modeled the SMLM data as a 3D point cloud^29–31^, a well-established representation used in 3D visual computing. Virtual connections between points transform the point cloud into a network modeled computationally as a graph with nodes (or vertices); edges are connections between nodes (points) that weight the distance between nodes. Such network representations have been widely and successfully adopted for analysis of brain, social and computer networks^32–36^. Using machine learning approaches, features that distinguish networks are identified and used to understand the underlying organization or architecture of the network. Here we apply point cloud network analysis to SMLM data sets to define the molecular architecture of plasma membrane-associated caveolae and caveolin-1 (Cav1) scaffolds.

Formation of caveolae, 50–100 nm plasma membrane invaginations, requires both the coat protein Cav1 and the adaptor protein polymerase I and transcript release factor (PTRF or CAVIN1)^37^. Cav1 is also expressed in functional non-caveolar domains, or Cav1 scaffolds, that cannot be distinguished from caveolae by fluorescence microscopy, as both are smaller than the diffraction limit^38^. Metastatic PC3 prostate cancer cells express Cav1 but no CAVIN1 and no caveolae; stable transfection of CAVIN1 (generating PC3-PTRF cells) induces caveolae^37^. Application of machine learning to point cloud SMLM network analysis of Cav1 distribution in PC3 and PC3-PTRF prostate cancer cells has now defined Cav1 localization signatures for scaffolds and caveolae.

## Results

### Network Analysis of 3D Cav1 Point Clouds

**Figure 1:**
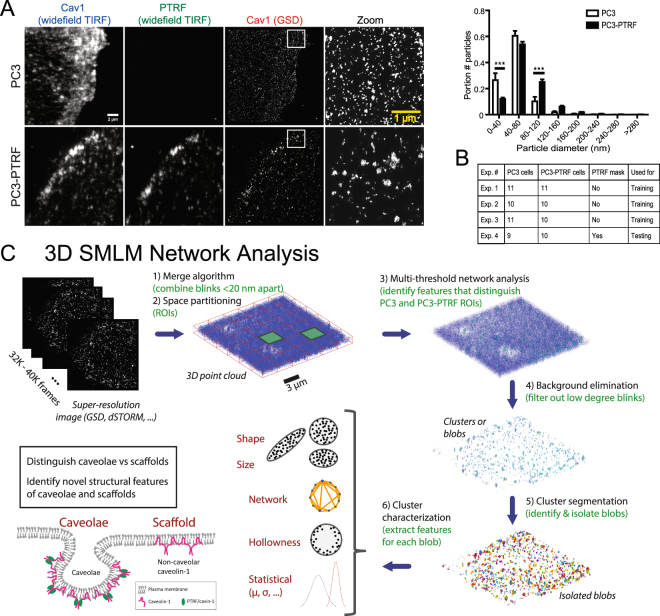
Computational network analysis for SMLM. (**A**) TIRF wide-field imaging of Cav1 and CAVIN1 and SMLM GSD imaging of Cav1 in PC3 and PC3-PTRF cells. Spot diameters from the two cell types (Experiment 1, see B) were binned (Bar: SEM; ***p < 0.001). (**B**) Details of 4 SMLM experiments imaged using Leica GSD microscope. (**C**) Methodological pipeline to discover signatures of different Cav1 domains: 3D SMLM Network Analysis. A GSD image event list is converted to a 3D point cloud. Blinks within 20 nm are merged and the 3D point cloud divided into ROIs for multi-threshold network analysis. Network measures filter the 3D point cloud to obtain clusters (blobs). Features are extracted for each blob and blob identification achieved via unsupervised learning methods.

Indirect immunofluorescence labeling of PC3 and PC3-PTRF cells with anti-Cav1 primary and Alexa647 conjugated secondary antibodies is shown by TIRF and GSD-TIRF SMLM (Fig. 1A). Quantification of SMLM images identifies larger spots in caveolae-containing PC3-PTRF cells (Fig. 1A). We obtained the 3D locations (event list) of individual Cav1 blinks from three training experiments (Fig. 1B) based on Gaussian analysis of the PSF using Leica GSD software. To study clusters or networks of representative Cav1 localizations, and not blinks, we assigned Cav1 localizations from the acquired blinks. We then applied complex network analysis to assign edges between predicted Cav1 localizations (nodes) and generate measures that define relationships between nodes. For instance, “degree” is a measure of the number of edges incident (connected) to a given node within a certain distance threshold.

The analysis pipeline (Fig. 1C), referred to as 3D SMLM Network Analysis, consists of six computational modules: 1) Merging algorithm: Iterative merging of all blinks within the 20 nm resolution limit of SMLM to generate nodes corresponding to predicted Cav1 localizations; 2) Space partitioning: Dividing the cell into regions of interests (ROIs) to facilitate whole network measure analysis via parallel high performance computing (HPC); 3) Multi-threshold network analysis: Constructing weighted and unweighted networks at various proximity thresholds to identify features and thresholds that differentiate PC3 and PC3-PTRF ROIs; 4) Background elimination: Using discriminative network measures to filter out noisy blinks relative to a random point distribution; 5) Cluster segmentation: Using the mean shift algorithm^39,40^ to identify distinct clusters (or blobs) and extracting features for each cluster; and 6) Cluster characterization: Defining distinct clusters via unsupervised learning methods in PC3 and PC3-PTRF cells.

**Figure 2:**
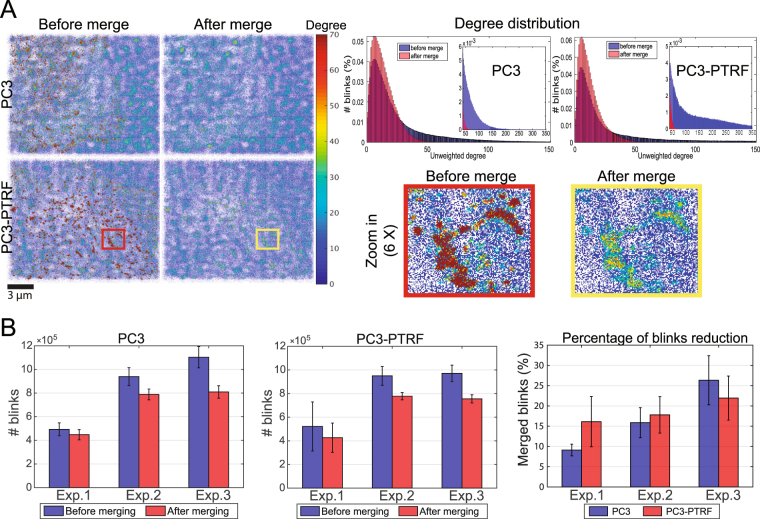
Iterative blink merging to correct multiple blinking of single fluorophores. (**A**) We utilized an iterative merging approach of blinks within T = 20 nm as a preprocessing step to correct for multiple blinking of a single fluorophore, blinking from multiple fluorophores on the same antibody, and multiple secondary antibody labeling of the same Cav1 molecule and associated drift. Network degree measure images and histograms of PC3 and PC3-PTRF cells before and after applying the merge module at 20 nm. (**B**) The number of blinks and the percent reduction of total blinks for each experiment following iterative merging at 20 nm. The error bars represent the standard deviation.

Multiple, temporally distinct blinks from the same fluorophore or same antibody-labeled Cav1 molecule can give rise to blinks with distinct localizations, thereby skewing the data and subsequent network analysis. To generate a robust method to remove high degree blinks and reconstruct a network of representative Cav1 localizations, we applied an algorithm that performs iterative merging of blinks within 20 nm, the reported resolution limit of the Leica GSD microscope. In short, for every blink, we find neighboring blinks within a 20 nm sphere centered around that blink. Starting with the blink with the largest number of neighbors, we replace that blink and all its neighbors by a new blink positioned at their average location. Then we continue to process the blink with the second largest number of neighbors and so on. We repeat the procedure until no pair of blinks are within 20 nm from each other. Note that we use k-d-tree search algorithm to speed up the process of finding the nearest neighbors. Running our algorithm takes <1 min to process an input point cloud of more than 1.5 million blinks. Application of the merging algorithm to PC3 and PC3-PTRF cells selectively reduces high degree (>50) nodes (Fig. 2A). Reduction in the total number of blinks by the merge algorithm varied from 9 to 26% (Fig. 2B).

### Statistical Analysis of Features at the ROI Level

To identify the proximity threshold of nodes in 3D space that best discriminates between PC3 and PC3-PTRF cells, we constructed weighted and unweighted undirected networks at thresholds from 20 nm to 250 nm. To enable high speed processing of the large data sets, up to 1 million blinks per image (Fig. 2B), we divided each image into 36 ROIs (3 × 3 × 1 *μm*^3^) and extracted 32 weighted and unweighted network features at 24 thresholds (i.e. 768 features/ROI; See Supp. Table S1 for a complete list of network features). We chose an ROI size of (3 × 3 × 1 *μm*^3^) to be significantly larger than the size of the biological structure we are studying, caveolae, of about 80–100 nm diameter and yet had manageable memory space.

**Figure 3:**
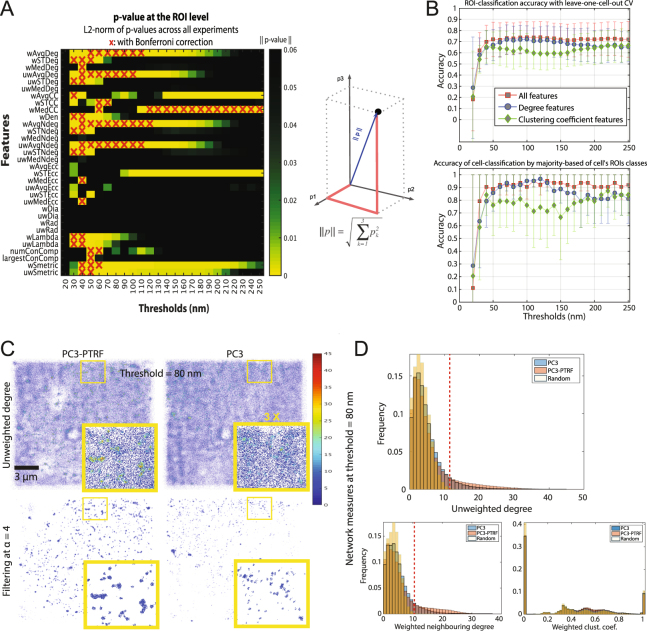
Multi-threshold analysis of PC3 and PC3-PTRF data at the ROI level. (**A**) Multi-threshold statistical analysis shows network measures and thresholds that discriminate PC3 and PC3-PTRF 3D point clouds. For each experiment, the p-values are calculated by the two-sided non-parametric Mann-Whitney statistical test to evaluate the null hypothesis that the network measures of the two populations (PC3 and PC3-PTRF) followed the same distribution. p-values (*p*_1_, *p*_2_, and *p*_3_) from Experiments 1–3 (see Fig. 1B) are aggregated via the L2-norm. We show the results with and without Bonferroni multiple comparisons correction. (**B**) Random decision forest accuracy of classifying the two populations at ROI (top) and cell level (bottom) using the whole feature set (red), degree features only (blue), and clustering coefficient features only (green). The error bars represent the standard deviation. (**C**) Filtering-out noisy blinks using unweighted degree at 80 nm via comparison with random graphs. (**D**) Filtering-out noisy blinks at 80 nm for unweighted degree, weighted neighboring degree (significant) and weighted clustering coefficient (non-significant). Vertical dashed red lines indicate level of noise removal. See Figs S2, S3.

We applied the nonparametric Mann-Whitney statistical test to find the features and corresponding thresholds at which they discriminate with statistical significance between PC3 and PC3-PTRF cells. Since we have a relatively large number of feature and threshold pairs, the chance of getting a significant p-value is high. We used Bonferroni multiple comparison correction to avoid getting significant features by chance. P value matrices (32 features ×24 thresholds) were combined by finding the L2-norm of *p*_1_, *p*_2_, and *p*_3_; feature-threshold pairs that survived the Bonferroni multiple comparison correction are marked with red X’s (Fig. 3A). We used L2-norm to combine the significance of the network measures over three different biological experiments. Any non-significant (large) p-value will dominate and make the resultant L2-norm non-significant. By doing so, we are being extra-conservative in the selection of features by ensuring that only if a feature is significant over the different experiments, will it be used with confidence in our analysis. Significant features grouped into two categories: degree features (wAvgNDeg, uwAvgNDeg, uwAvgDeg, wAvgDeg) and clustering coefficient features (wAvgCC and wMedCC). Degree features are more significant from 40–120 nm (average 80 nm) and clustering coefficient features from 120–250 nm (average 180 nm).

Random decision forest^41^ machine learning classification, using a leave-one-cell-out cross-validation (ROIs from each cell are excluded from training and used only in testing) strategy, tested the feature and threshold ranking. We report the classification accuracy of the detected class of (i) each test ROI and (ii) the cell, chosen to be the detected class of the majority of the ROIs (Fig. 3B). For both cases, classification accuracy is best for thresholds above 50 nm and increases for degree features between 70 and 130 nm (blue curve) and for clustering coefficient features between 170 and 250 nm (green curve) (Fig. 3B). Two peaks in the degree features histogram suggest the presence of low degree or noisy blinks and high degree or clustered nodes (Fig. S1).

Significant differences between PC3 cells, lacking caveolae, and PC3-PTRF cells, that have caveolae, were detected for degree features between 40–120 nm (average 80 nm) and for clustering coefficient features between 120–250 nm (average 180 nm). We therefore chose 80 and 180 nm thresholds for further analysis. To filter out noisy blinks and return clusters in the PC3 and PC3-PTRF data, we used degree features at threshold 80 nm (the mean of the significant thresholds) and compared network measures with those of a random graph (Fig. S2). The *node* is retained (i.e. not filtered out) if its degree value (*δ*_*i*_) is greater than the average degree value of a random graph (*δ*_*rand*_) multiplied by a scalar *α*, i.e. if ${\delta }_{i} > \alpha \times mean\,({\delta }_{rand})$. The parameter *α* is user-controlled to determine the extent of removal of noisy blinks (Fig. 3C,D). We compared two degree (uwAvgDeg; wAvgNDeg) and one clustering coefficient (wAvgCC) measures at thresholds 80 and 180 nm (Figs 3D and S3) with network measures of a random graph. We tuned *α* so that *δ*_*i*_ does not exceed the histogram tail of the random graph degree features (dashed red line) and obtained the best filtration with *α* = 4 (Fig. S3). Degree feature filtering worked better at threshold 80 nm, with wAvgNDeg providing stricter filtering that removes tiny clusters. Filtering eliminates background labeling but also monomeric Cav1 nodes, such that our analysis selectively includes Cav1 clusters or blobs.

### Blob Segmentation, Grouping and Matching

Having removed high degree blinks and filtered-out noisy blinks, we then employed the mean-shift algorithm^39,40^ to segment and separate each blob. Twenty-eight measures at 80 nm threshold describing size, shape (spherical, planar, linear) and topology (hollowness, modularity) were extracted for each individual blob (Supp. Table S2). Some of the features were designed particularly for this application (e.g. Hollowness), others are standard for describing network (e.g. network features), while a third type is a set of features that are common in other fields and that we leveraged in our application. Our features describe every 3D point cloud blob by a low-dimensional descriptor that can be used to discriminate the blobs using the machine learning approaches. In Supp. Table S2, we categorize the used measures into shape, hollowness and network features and cite published works related to the features so the reader can find the detailed descriptions, e.g. equations, for the used features. Notice that we have two groups of features. Features at the ROI level are summarized in Supp. Table S1 and at the blob level summarized in Supp. Table S2. The first group is comprised of 32 network measures extracted for every ROI at various proximity thresholds (24 thresholds). These features are used to filter out the noisy blinks and retain the biological clusters/blobs. The second group of features is used as a blob descriptor. We extracted 28 features of every blob to be used for the blob identification step.

**Figure 4:**
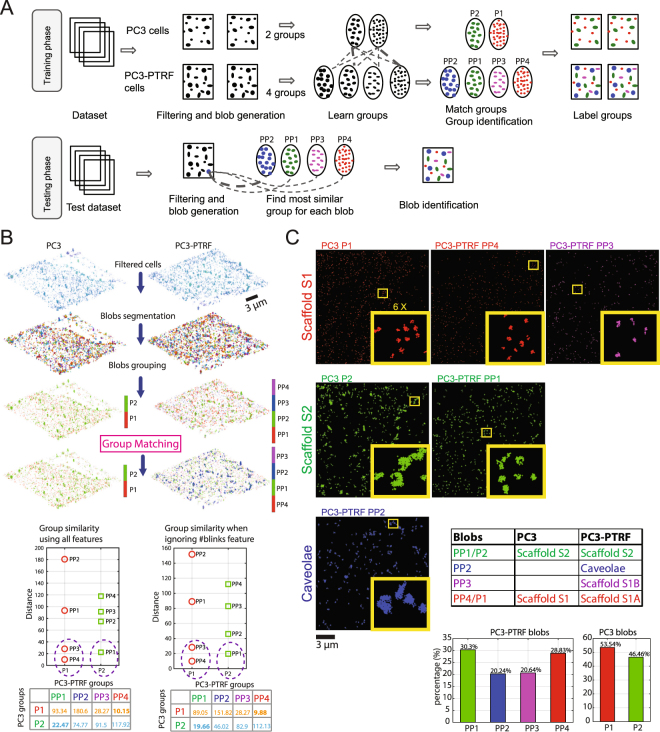
Unsupervised learning identifies different blobs. (**A**) The unsupervised learning framework to build the blob identification model based on datasets. Training phase: we used the cells from both populations of the first three experiments (Fig. 1B) to build the learning model using the unsupervised clustering. The cells are divided into ROIs. A multi-threshold network analysis for each ROI is employed to filter-out the noisy blinks and find the clustered nodes. The blobs are generated from the clustered nodes using the mean shift algorithm. A new set of features are extracted from each blob and fed into the unsupervised clustering (X-means) to learn the different groups. The groups from PC3 and PC3-PTRF populations are matched using the similarity analysis to identify the groups’ types. The matched groups are used to label the blobs on the cells. Testing phase: we used the built model to identify the blobs of the cells from experiment 4. The cell is passed via the space division to get the ROIs. The multi-threshold analysis is applied to filter-out the noisy blinks and return the clustered nodes. The blobs are generated using the mean shift algorithm. The same set of features is extracted for each blob. Each blob feature vector is tested against the centroid feature vector of the learned groups. The closest distance is the most similar group to this blob. The blobs are finally labeled based on the similarity of their feature vector with groups’ centroids. (**B**) After filtering, blob-level feature analysis and segmentation identifies 2 groups (P1, P2) in PC3 and 4 groups (PP1, PP2, PP3, PP4) in PC3-PTRF by unsupervised learning. One-to-one group matching (box) with distances among feature vector of groups centers used as the similarity measure (closer groups are more similar). (**C**) Each group of blobs (S1 or S2 scaffolds or caveolae) is extracted and shown as different channels. Graph shows percent distribution of blobs in PC3-PTRF and PC3 cells.

The X-means^42^ (unsupervised clustering algorithm) returned the optimal number of groups in each population: 2 groups (P1, P2) for PC3 and 4 groups (PP1, PP2, PP3, and PP4) for PC3-PTRF. Unsupervised clustering methods gathered similar blobs in the same group and dissimilar blobs in different groups (Fig. 4A). To match the groups, we calculated the Euclidean distances between every pair of group centers, a vector of 28 features, in a distance matrix (Fig. 4B). By setting the similarity threshold *β* to 30, we found the most similar groups across the two populations. The PC3 P1 group is most similar to PC3-PTRF PP3/PP4 (S1 scaffolds) and P2 most similar to PP1 (S2 scaffolds). The PC3-PTRF group most dissimilar to PC3 P1 and P2 groups is PP2, suggesting that it corresponds to caveolae. Identical group matching was obtained if the estimated number of molecules feature was not included and only the remaining 27 features were used (Fig. 4B). Representative images of the blob groups and their abundance in PC3-PTRF and PC3 cells are shown in Fig. 4C.

### Blob Signatures

**Figure 5:**
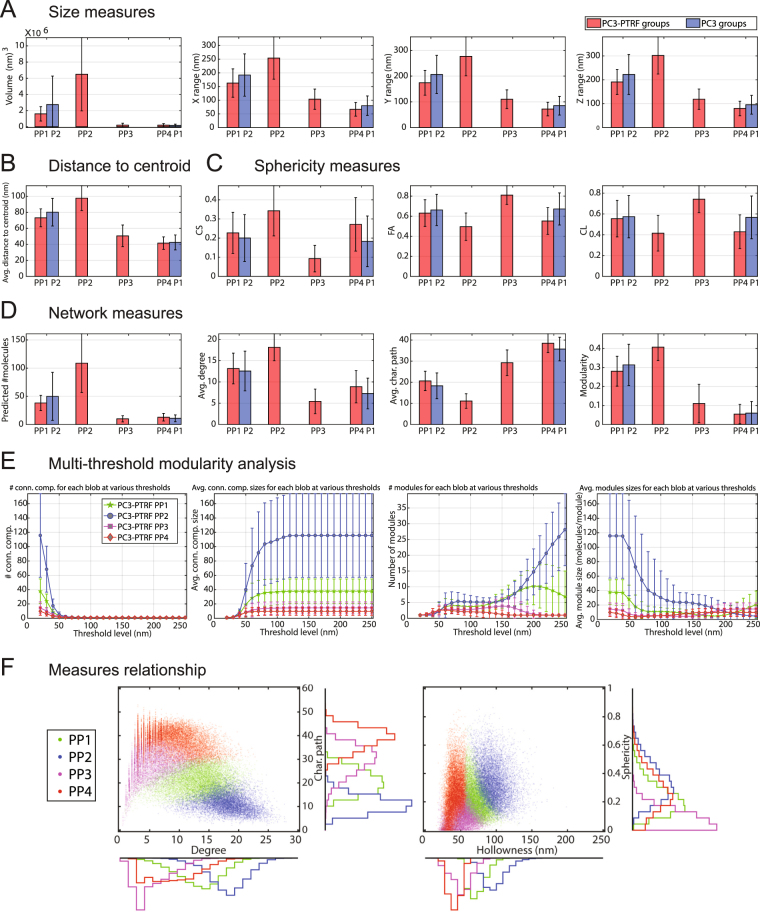
Digital bio-signatures of caveolae and scaffolds. Of 28 signatures, we present measures for each class of blob: (**A**) Size measures (volume, X-, Y-, Z-range); (**B**) Hollowness measure: (distance to centroid); (**C**) Shape measures (sphericity, fractional anisotropy, linearity); (**D**) Network measures (# predicted molecules, degree, character path, modularity). (**E**) Multi-threshold modularity analysis shows the number and average size of connected components and modules for PC3-PTRF blobs at different thresholds. (**F**) 2D relationship between features for different PC3-PTRF blobs are shown. The error bars represent the standard deviation. For (**A**–**D**), the differences for every pair of the groups are statistically significant (p < 0.0001).

PP2 blobs or caveolae are larger (∼250–300 nm) than S1 (P1/PP3/PP4) and S2 (PP1/P2) scaffolds (Fig. 5A). S2 scaffolds are larger than S1 scaffolds and their increased height suggests that S2 scaffolds present a non-planar morphology (Fig. 5A). PP2 caveolae are the hollowest structures with an average distance to centroid of ∼95 nm and no nodes <60 nm from centroid (Fig. 5B,F). For S1 scaffolds, the minimum distance to centroid was 20 nm, indicating that these structures are filled in. S2 scaffolds were also hollow, showing an average distance to centroid of 70 nm and no nodes <50 nm from centroid (Fig. 5B,F). PP2 caveolae contain 109 ± 52, S1B (PP3) scaffolds 10 ± 6, S1A (PP4) scaffolds 13 ± 7 and S2 (PP1) scaffolds 38 ± 14 nodes, corresponding to predicted number of Cav1 molecules (Fig. 5D). S1 scaffolds therefore correspond to SDS-resistant oligomerized Cav1 units of 15 Cav1 molecules^43^ and PP2 blobs approach the predicted 144 ± 39 Cav1’s per caveolae^44^. S2 scaffolds are an as yet unidentified non-planar Cav1 scaffold.

PP2 caveolae show the highest degree, lowest clustering coefficient and shortest characteristic path length and have higher modularity (Fig. 5D). We employed the Newman method^45^ to find the optimal number of modules and extract modularity features for each blob at thresholds from 20 to 250 nm. Most of the blobs become one connected component (i.e. all the nodes are connected) at thresholds >50 nm and show a stable modular structure between thresholds of 60–140 nm, with PP2 caveolae presenting 5–6 modules per blob (Fig. 5E). Representative measures relationship analysis plots for degree vs character path and for hollowness vs sphericity show clear clusters for the four blob groups (Fig. 5F).

**Figure 6:**
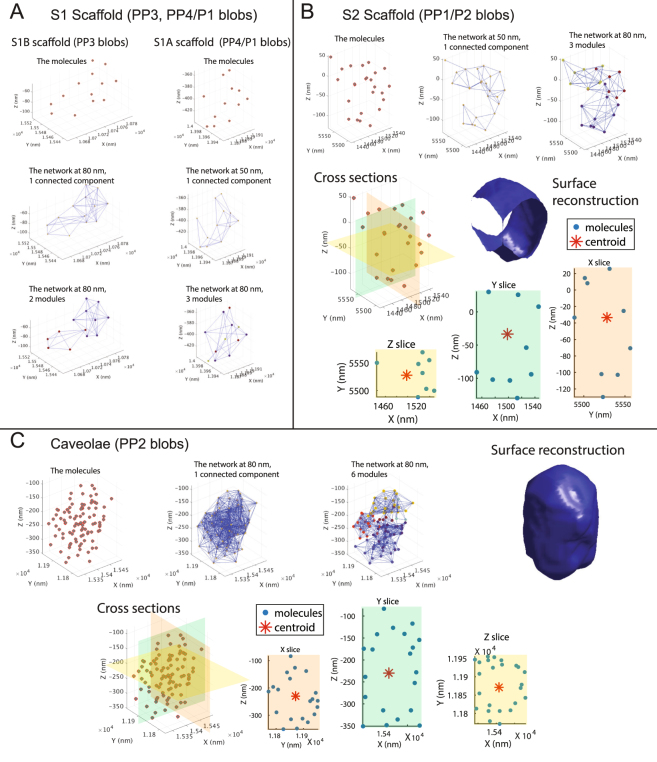
Visualization of representative blobs from PC3/PC3-PTRF cells. Blob’s molecules and networks (including number of connected components and modules) at various thresholds are shown for: (**A**) S1 scaffolds (PP3/PP4 blobs); (**B**) S2 Scaffolds (PP1 blob); and (**C**) Caveolae (PP2 blob). Cross-sections, XYZ slices and surface reconstructions are shown for S2 scaffolds and caveolae. Slice thickness for S2 scaffolds is 20% of the whole range of the blob size in each dimension and for caveolae 10%. S2 scaffolds are hemi-spherical and consist of 3-4 modules. Caveolae are spherical and hollow and consist of 5-6 modules.

For selected S1, S2 scaffolds and caveolae, predicted 3D molecular distribution, network connections at the lowest threshold at which connected components equals one, and modular structure are shown in Fig. 6 (See animated rotating structures in Supp. Videos S1–4). S1A (P1/PP4) and S1B (PP3) scaffolds are composed of 10–15 molecules, equivalent to SDS-resistant Cav1 oligomers^43^ and differ primarily in that S1B scaffolds are more elongated and S1A more spherical. The presence of pentagonal sub-modules supports EM data of polygonal repeating units in the caveolar cage^46^. PP1/P2 S2 scaffolds present a hemi-spherical shape and hollow core from XYZ cross-sections (Fig. 6B). PP2 caveolar blobs form highly interconnected hollow, spherical networks that contain, for the representative blob shown here, 6 modules (Fig. 6C).

**Figure 7:**
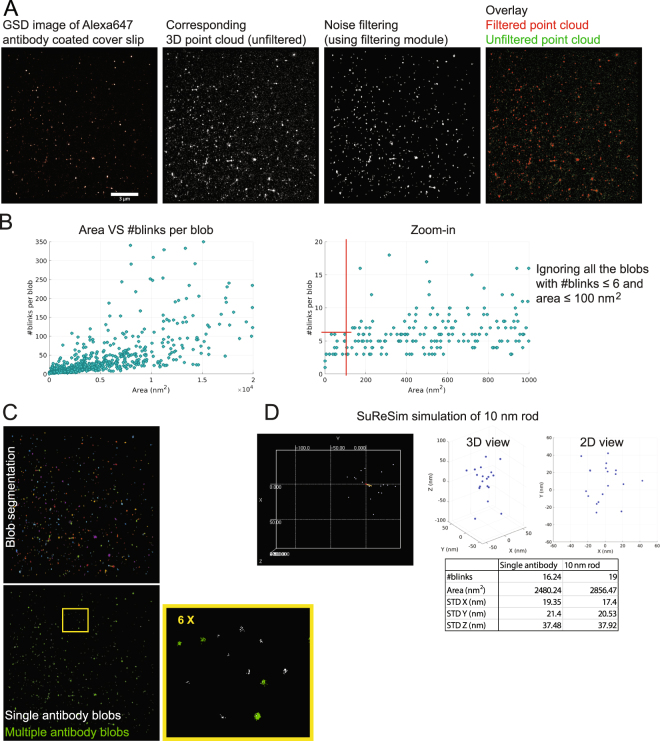
Experimental single antibody blinking data matches simulated data obtained for a 10 nm rod using SuReSim software. (**A**) A super-resolution GSD image of a cover slip coated with Alexa647-conjugated anti-rabbit secondary antibodies was imaged and the GSD image, unfiltered 3D point cloud and filtered point cloud, processed using filtering module 4 of the 3D SMLM Network Analysis pipeline (Fig. 1C) to retain blobs and remove noisy blinks, are shown. (**B**) Segmentation module 5 (Fig. 1C) was applied to the data to obtain the individual blobs and plots of #blinks per blob vs blob area are shown. Blobs with area ≤100 nm^2^ all had fewer than 6 blinks (red lines) and we removed all blobs with fewer than 6 blinks and with area ≤100 nm^2^. (**C**) Single and multiple antibody blobs were identified using K-means clustering (K = 2) based on the extracted blobs’ features. By identifying the single antibody blobs, we derive the blinking data from an isolated protein experimentally. (**D**) We used the derived blinking data from the real single antibody blobs to image a rod of 10 nm length using SuReSim software^47^. The table shows the average standard deviation for the spread of the blinks from the blobs for the experimental data derived from a single antibody and for the simulated (SuReSim) imaging of a 10 nm rod.

To assess whether the approach used could identify single molecules, we coated cover slips with Alexa647-conjugated anti-rabbit secondary antibodies. The image obtained using the same Leica GSD microscope that we used to image the PC3 and PC3-PTRF data, the resultant point cloud and filtered point cloud, removing background, are shown in Fig. 7A. The blobs are then segmented to get individual blobs and dimensions of every segmented blob are then extracted. Area vs #blinks analysis of blobs in the filtered point cloud highlights the presence of small clusters of area less than 100 nm^2^ that contain at most 6 blinks (Fig. 7B). These clusters were removed from the analysis. Changing #blinks and area parameters 6 and 100 nm^2^ by ± 20% (tested {4, 5, 6, 7, 8} blinks and {80, 90, 100, 110, 120 nm^2^} areas) did not affect the result; changes between the various conditions analyzed were <5% with no statistically significant difference (i.e. p ≫ 0.05). To differentiate the single antibody blobs from the blobs derived from antibody clusters, we utilized K-means clustering (K = 2) based on the extracted blobs’ features (Fig. 7C). We then determined the average standard deviation for the spread of blinks for single antibody blobs (X = 19.35, Y = 21.40, Z = 37.48 nm) and antibody cluster blobs (X = 27.24, Y = 30.92, Z = 70.08 nm).

SuReSim software simulates imaging data of ground-truth structures with any 3D geometry using specific antibody labelling and acquisition parameters^47^. We used the labeling and acquisition parameters from single antibody SMLM labeling to simulate SMLM imaging of a rod of 10 nm length, corresponding to a single antibody, using SuReSim (Fig. 7D). A representative blob including 2D and 3D views are shown. Blob dimensions and #blinks per blob for both the real data analysis of single antibody labeling and the SuReSim simulation of a 10 nm rod closely match and suggest that clusters obtained from single antibody labelling generates a 20 × 20 × 40 *nm*^3^ point cloud.

**Figure 8:**
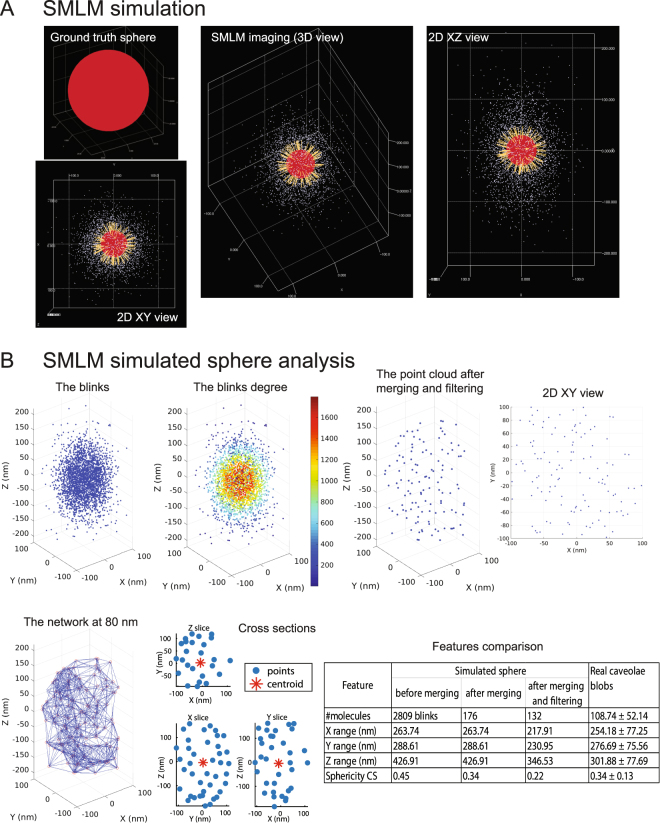
Network analysis of a simulated 60 nm sphere using SuReSim software. (**A**) A ground truth sphere that mimics caveolae-like structures with 60 nm diameter appears in red was used to obtain an SMLM event list using SureSim software using labeling and acquisition parameters experimentally derived from single antibody blinking data (Fig. 7). The yellow bars represent the epitopes and the white points the acquired blinks. In the SuReSim software simulation, we set the lateral precision (XY) to be 20 nm and the axial precision (Z) to be 38 nm and the epitope density to be 0.012821 nm^−2^ with labeling efficiency of 100% to match the reported 145 Cav1 molecules per caveolae^57^. 3D and 2D views of the imaged point clouds show increased Z spread, reflecting the poorer Z resolution relative to XY resolution. (**B**) Analysis of biological signatures of the simulated SMLM data of a 60 nm sphere relative to the real SMLM-imaged caveolae. We analyzed the simulated data using the same pipeline (Fig. 1C) used to analyze the real data. We calculated the network degree for the blinks before merging then preprocessed the data by merging the blinks at 20 nm and, then, filtered out the noisy blinks. The resultant point clouds are shown in 3D and 2D views. The dimensions of the point clouds are similar to the bio-signatures of the caveolae blobs that we obtained for real data (Fig. 5A–D and Fig. 6C).

We then used SuReSim software to simulate SMLM imaging data for a caveolae-like structure. Using the same labeling and acquisition parameters as for the 10 nm rod SuReSim simulation (Fig. 7D), we generated an SMLM point cloud for a 60 nm sphere, mimicking a caveola (Fig. 8A). We applied our analysis pipeline to the simulated data and obtained results (number of predicted molecules, shape, and size features) very similar to what we obtained for real data of caveolae structures (Fig. 8B). Future analyses combining improved labeling approaches (such as smaller Fab antibody fragments), drift-controlled acquisition and cluster analysis should lead to substantially improved resolution and better definition of molecular architecture.

## Discussion

Application of computational network modelling and machine learning based 3D pattern analysis tools to SMLM data sets (3D SMLM Network Analysis) has identified plasma membrane-associated Cav1 scaffolds that combine to form caveolae and larger scaffolds. S1A scaffolds are equivalent to the 200–600 kD SDS-resistant, 8 S Cav1 oligomers of 14-15 Cav1 molecules^43,48–50^. The presence of these structures in our analysis indicates that they can be delivered to the cell surface without assembling into larger structures in the Golgi^50^. S1A scaffolds dimerize to form S1B scaffolds and groupings of 3-4 S1A scaffolds form larger hemi-spherical S2 scaffolds. Biochemical fractionation using sucrose density gradients and mild lysis conditions reported, in addition to 8 S complexes, larger 70 S complexes, predicted to contain 160 Cav1 molecules and corresponding to Cav1 oligomers of assembled caveolae^50^. Both S1B and S2 scaffolds therefore represent intermediate Cav1 oligomers, that may be unstable upon detergent extraction. Regrouping of S1A scaffolds to form larger scaffolds and caveolae is associated with a more compact Cav1 organization (higher degree, reduced characteristic path) and increased curvature (sphericity). The presence of hemispherical S2 scaffolds in PC3 cells suggests that Cav1 oligomerization can induce membrane curvature independently of CAVIN1, as reported for Cav1 in bacteria^46^.

Consistently, 10 Cav1 densities and a decagonal outline has been reported for Cav1-induced caveolae in bacteria^46^. Further, EM tomography of caveolae labeled with MINISog, reports the presence of 12 regularly spaced Cav1 densities with a half-maximum width of 8–10 nm and spacing of 10 nm^51^. A recent model of caveolae based on biochemical and cryoEM analysis proposed that caveolae are polygons (dodecahedrons) of Cav1 disks, equivalent to 8 S oligomers^52^. Our identification of 6-7 caveolae modules in the caveolar coat is consistent with this model and suggests that 8 S oligomers, or S1A scaffolds, regroup to form larger scaffolds that combine to form a caveolae (Fig. 6C). The striated caveolar coat observed by deep-etching EM and platinum coating^53^ has been suggested to reflect the presence of rod-like cavin oligomers^51,54,55^, that may form a net around the Cav1 disks^52^.

Using blinks to define molecular architecture assumes that a blink equals a molecule. However, the same fluorophore can blink twice in succeeding acquisition frames^56^ and the same molecule can be labeled by different fluorophores, either on the same secondary antibody or on different secondary antibodies binding to the same protein. To address these possibilities, we developed an iterative merging algorithm, in which all blinks within a specified distance (the merging threshold) are combined into one average position. The algorithm iteratively combines all blinks within the merging threshold, in this study set at 20 nm, and converges when there is no pair of blinks within the merging threshold. As such, the merging algorithm allows the interrogation of molecule number and organization within the structure.

As SMLM resolution approaches molecular distances between proteins, and particularly at high protein densities, the blinking cycles of individual fluorophores overlap in time such that undersampling and undercounting is a major concern^21^. Indeed, our approximation of 110 Cav1 molecules is less than the predicted 145 Cav1 proteins per caveolae or 160 Cav1’s per 70 S oligomer^50,57^. We used a fixed merge threshold for this study that did not take into consideration the differential Z resolution of cylindrical lens-based SMLM. Indeed, analysis of single Alexa647-conjugated antibodies coated on a cover slip generated point clouds with approximate XY standard deviations close to 20 nm and Z dimension close to 40 nm. Future studies should consider modulation of the merge threshold to adapt to labeling conditions, microscope acquisition parameters and the different Z resolution of standard SMLM systems.

The ∼250 nm average diameter of caveolae blobs is substantially larger than the size of caveolae; although minimum values (X-range: 89.4 nm; Y: 86.8 nm; and Z: 78.2 nm) of the 80,000 blobs analyzed approach the 60–80 nm caveolae diameter by EM^58^. Consistent with our findings, EM analysis of anti-caveolin labeling is cytoplasmic and at a distance from the membrane^51^. In addition, our analysis of simulated SMLM data from a 60 nm sphere, resembling a caveolae, generated point cloud distributions of similar sizes to those we observed experimentally for real caveolae (Fig. 8). Similarly, the data obtained by SuReSim simulation of a 10 nm rod closely matched that of experimentally derived point clouds for isolated Alexa-647 conjugated antibodies (Fig. 7D). Together, these analyses validate the obtained bio-signatures of the different structures using the parameters of the 3D SMLM Network Analysis pipeline.

Importantly, we are the first to use machine learning algorithms to quantify specific features of blobs and identify them automatically. We equipped 3D SMLM Network Analysis with a feature extraction module that can be used to extract features for individual clusters. The clusters features can be used for further analysis (e.g. retrieval, identification, grouping, molecular architecture analysis, etc. of the clusters) by machine learning. Network analysis, as described here, can therefore be used to reconstruct heterogeneous clusters of different shapes and sizes. Further development and refinement of this approach should allow the determination of the molecular architecture of multiple sub-diffraction limit cellular structures. As indicated, 3D SMLM Network Analysis is a fast, 3D, scalable, and multi-scale method. Moreover, SMLM Network Analysis is enabled with single cluster visualization and can be integrated with different machine learning approaches to extract and automatically identify cluster features.

Network analysis has therefore provided us with invaluable insight into the structure of sub-diffraction limited Cav1 domains. This analysis has defined three classes of non-caveolar scaffolds, including a heretofore undescribed hemispherical intermediate distinct from the biochemically defined 8 S and 70 S Cav1 complexes^50^ and enhanced our understanding of how Cav1 molecules organize to form a caveolae. Indeed, we argue that point cloud network analysis is particularly appropriate for the analysis of the blink distributions generated by SMLM allowing super-resolution fluorescence microscopy to define the molecular structure, stoichiometry and architecture of macromolecular complexes.

3D SMLM Network Analysis is, therefore, an integrated pipeline that consist of various computational modules for 3D SMLM quantification and analysis. Each module can be replaced by an equivalent module based on state-of the art methods and re-integrated into the pipeline. The current pipeline can be further improved and we are currently optimizing the implementation such that we can handle the whole cell as one big-ROI. Some other improvements that can be added to the current pipeline are the merging, denoising, and segmentation modules. The merging module may undercount/overcount. The current denoising module can remove signal (not noise). In addition, the segmentation module may over-segment or under-segment and may combine nearby structures into one. Finally, the detection of the different domains depends on the extracted network measures and features, which in turn depend on the different artifacts of the aforementioned modules. We do not believe that the current merge algorithm can distinguish between an active dye that blinks multiple times and multiple fluorophores on a single secondary antibody. Indeed, we consider that such blinks all contribute to the non-biological networks and our goal is not to distinguish these blinks but rather interpret them to determine the molecular localization that gave rise to these non-biological blinks.

## Materials and Methods

### GSD SMLM Imaging

PC3 and PC3-PTRF cells were cultured in RPMI-1640 medium (Thermo-Fisher Scientific Inc.) complemented with 10% fetal bovine serum (FBS, Thermo-Fisher Scientific Inc.) and 2 mM L-Glutamine (Thermo-Fisher Scientific Inc.). All cells were tested for mycoplasma using PCR kit (Catalogue# G238; Applied Biomaterial, Vancouver, BC, Canada). Cells were plated on cover slips (NO. 1.5 H; coated with fibronectin) for 24 h before fixation. Cells were fixed with 3% paraformaldehyde (PFA) for 15 min at room temperature, rinsed with PBS/CM (phosphate buffered saline complemented with 1 mM MgCl_2_ and 0.1 mM CaCl_2_), permeabilized with 0.2% Triton X-100 in PBS/CM, and blocked with PBS/CM containing 10% goat serum (Sigma-Aldrich Inc.) 1% bovine serum albumin (BSA; Sigma-Aldrich Inc.). Then the cells were incubated with the primary antibody (rabbit anti-caveolin-1; BD Transduction Labs Inc.) for 12 h at 4 °C and with the secondary antibody (Alexa Fluor 647-conjugated goat anti-rabbit; Thermo-Fisher Scientific Inc.) for 1 h at room temperature. The primary and secondary antibodies were diluted in SSC (saline sodium citrate) buffer containing 1% BSA, 2% goat serum and 0.05% Triton X-100. Cells were washed extensively after each antibody incubation with SSC buffer containing 0.05% Triton X-100. Post-fixation was applied using 3% PFA for 15 min followed by extensive washing with PBS/CM. For single antibody cover slip coating, cover slips were incubated with 2.5 μg fibronectin for 12 h at 4 °C and then with Alexa647-conjugated anti-rabbit secondary antibody diluted 1:500 in PBS/CM for 1 h at room temperature.

Before imaging, the imaging buffer was freshly prepared with 10% glucose (Sigma-Aldrich Inc.), 0.5 mg/ml glucose oxidase (Sigma-Aldrich Inc.), 40 μg/mL catalase (Sigma-Aldrich Inc.), 50 mM Tris, 10 mM NaCl and 50 mM β-mercaptoethylamine (MEA; Sigma-Aldrich Inc.) in double-distilled water^2,56^. The cells were immersed in the imaging buffer and sealed on a glass depression slide.

GSD super-resolution imaging was performed on a Leica SR GSD 3D system using a 160 × objective lens (HC PL APO 160 × /1.43, oil immersion), a 642 nm laser line and a EMCCD camera (iXon Ultra, Andor). Full laser power was applied to pump the fluorophores to the dark state, and at frame correlation value 25% the imaging program auto-switched to acquisition with 50% laser power. Epi-illumination was applied for the pumping process while a TIRF illumination with 100-nm penetration depth was applied for the acquisition step. For cells, acquisition was done for 5 min with camera exposure time at 6.43 ms/frame and for single antibody coated cover slips for 13 minutes with camera exposure time at 20 ms/frame. The event lists were generated using the Leica SR GSD 3D operation software with XY pixel size 20 nm, Z pixel size 25 nm and Z acquisition range +/−400 nm.

### Computational Methods

#### Graph Construction

Each image has dimensions of 18 × 18 × 1 *μm*^3^. For analysis, each cell is divided (tiled) into 36 ROIs of 3 × 3 × 1 *μm*^3^. Each ROI is much greater than subcellular structures. Tiling the cell into ROIs is essential to speed up processing time for the whole cell via HPC cluster computer.

We represent locations (X,Y,Z) of Cav1 event lists generated by Leica GSD analysis software as a point cloud (a set of points in 3D space), a well-established representation used in 3D visual computing^29–31^. An iterative blink merging algorithm merges blinks within 20 nm of each other, the resolution limit of the GSD approach, removing high degree (high interacting) points (i.e. from the same fluorophore or clusters of fluorophores). Virtual connections constructed between the resulting 3D points transform the Cav1 point cloud into a network modeled computationally as a graph with: nodes (representing a single Cav1 protein); edges (capturing the interaction between pairs of proteins); and weights (encoding the distances between nodes, up to an upper threshold limit). We calculate network measures (see Table S1 for list of network measures) at multiple distance thresholds to identify measures that discriminate between caveolae-containing PC3-PTRF and PC3 cells lacking caveolae. With hundreds of thousands to millions of Cav1 localizations (i.e. network nodes), we partitioned the imaged 3D space using equal-sized 3D tiles and distributed parallel computations across many nodes of our HPC clusters. A filtering step retained only the sub-networks of nodes or blobs whose properties are different from those of randomly generated networks (Fig. S2).

Well-established, quantifiable measures can then be extracted from these networks (Clustering Coefficient; Node Density; Connected Components; Degree; Closeness and Betweenness; Small Worldness; etc.) and divided into different classes: (spatially) global vs. local; microscopic (node-level) vs. macroscopic (graph-level) vs. mesoscopic (community-level); network integration vs. segregation; geometrical vs. topological, etc.^35^. Using machine learning approaches, features distinguishing networks can be identified and used to understand the underlying organization or architecture of the network.

We represent the nodes (i.e. predicted Cav1 molecular localizations) as graphs (networks), where a graph *G* = (*V*, *E*) is a pair of set of vertices or (nodes) *V* and set of edges *E*, where $|V|=n$, $|E|=m$, *n* is the number of nodes, and *m* is the number of edges. An edge is connected between a pair of nodes (*i*, *j*) ∈ *V* if the Euclidean distance between the nodes ${d}_{ij}\le T$, where *T* is the proximity threshold. The Euclidean distance ${d}_{ij}={d}_{ji}=||{C}_{i}-{C}_{j}|{|}_{2}$, where C_i_ is the 3D coordinate of point *i* (the interaction between node *i* and node *j* is symmetric), and hence the graph is undirected. We constructed two types of undirected graphs, weighted and unweighted. In the weighted undirected graph, the edge weight is set as ${w}_{ij}=\frac{1}{{d}_{ji}}$, i.e. stronger edges (higher weights) connect more proximate nodes.

### Modularity Analysis of the Blobs

We employed the Newman method^45^ to find the optimal number of modules (or communities) in a given network. The modules were extracted using the eigenvectors decomposition from the adjacency matrix that represents the blob’s network. The modularity is one of the network measures that we used to estimate the intra-community similarity and inter-community dissimilarity of the molecules in one blob. If the modularity measure is very high, this means that the blob consists of modules (or communities, sub-clusters), wherein each module is a highly intra-connected set of molecules.

### Group Matching

To match groups of blobs (i.e. hypothesized as being of the same type) from the population of PC3 cells to similar groups in PC3-PTRF cell population, we first form an *N* × *M* similarity (or affinity) matrix *Y*, in which high similarity between the groups is encoded with small distances. *N* is the number of groups in the first population (PC3) and *M* is the number of groups in the second population (PC3-PTRF). The entry in the *i* − *th* row and *j* − *th* column of *Y* is set to ${Y}_{ij}={e}^{-||{y}_{i}-{y}_{j}|{|}_{2}}$, where *y*_*i*_ and *y*_*j*_ are the feature vectors (Table S2) describing the centroids of *group*_*i*_ in *population*_1_ and *group*_*j*_ in *population*_2_, respectively, and $||{y}_{i}-{y}_{j}|{|}_{2}$ is the Euclidean distance. Then, we match *group*_*i*_ in *population*_1_ with the ${j}^{\ast }-th$ group in *population*_2_, where ${j}^{\ast }=argmi{n}_{j}\,{Y}_{ij}$. We apply a matching threshold *β* to set the minimum value of similarity between the matched groups: If *min*_*j*_ *Y*_*ij*_ > *β* for all *j* groups, then the *group*_*i*_ is not matched to any of the *j* − *th* groups in *population*_2_. Note that more than one similar group could be returned.

### Blink Filtering by Leveraging Random Graphs

To decide whether or not a blink, in an ROI of a real cell, is regarded as noisy and hence must be filtered out, we resort to comparing the network properties of that node representing the blink with the corresponding properties of nodes of a purely random graph. Those nodes with properties similar to those of the purely random graph are regarded as random noise (and filtered out) and the remaining nodes are retained. The random graph is constructed with the same number of nodes as in the real cell ROI, and each spatial coordinate of the location of its nodes is generated with same distribution of the cell ROI blinks. For example, the locations of the blinks in both the X and Y dimensions followed a uniform distribution, while the distribution of the blinks in the Z dimension followed a normal (Gaussian) distribution. An example of generated random blinks that correspond to a real ROI taken from one of the PC3-PTRF cells in our data set (Fig. S2).

### SMLM Simulation Using SuReSim Software

We used the recently published SuReSim software^47^ to simulate SMLM imaging for single molecule (i.e. a 10 nm rod resembling a single antibody) and a sphere with diameter of 60 nm to resemble a caveolae. We used the average standard deviation for blinks from the single molecule clusters to find the localization precision values. We set the lateral precision (XY) to be 20 nm and the axial precision (Z) to be 38 nm. We recorded 40,000 frames and we set the label epitope distance to be 20 nm and the epitope density to be 0.012821 nm^−2^ with labeling efficiency of 100% to match the reported 145 Cav1 molecules per caveolae^57^. All the SuReSim simulation experiments were performed using the direct simulation workflow. The SureSim simulated data event list was processed using 3D SMLM Network Analysis and the obtained bio-signatures were compared with real data obtained for caveolae in PC3-PTRF cells.

## Electronic supplementary material

Supplemental Material

Supp. Video S1

Supp. Video S2

Supp. Video S3

Supp. Video S4
