# Supplementary material for: Super Resolution Network Analysis Defines the Molecular Architecture of Caveolae and Caveolin-1 Scaffolds
Source: Sci Rep. 2018 Jun 13;8:9009. doi: 10.1038/s41598-018-27216-4 (PMC5998020; doi:10.1038/s41598-018-27216-4)
Supplement: Supplementary file 1 — Supplemental Material [file 41598_2018_27216_MOESM1_ESM.pdf]

Super Resolution Network Analysis Defines the Molecular Architecture of Caveolae and Caveolin-1 Scaffolds

Ismail M. Khater[1], **Fanrui Meng**[2], **Timothy H. Wong**[2], **Ivan Robert Nabi**[2*#], **Ghassan Hamarneh**[1*]

SUPPLEMENTAL TABLE LEGENDS

Supp. Table S1. The network measures used in this work. For more details about the definitions of measures and their formulae, we refer the reader to [1,2]**.** Also see Figure 1 and Figure 3A.

Supp. Table S2. The set of features extracted for every blob. The "nodes" in the description below refers to the predicted localizations of Cav1 molecules of each blob. For the network features we refer the reader to [1,2]. For the shape feature (FA, CL, CS, and CP) we refer the reader to [3]. We designed the rest of the features. Also see Figure 4 and Figure 5. The list of features was designed to span a set of categories of features: shape, geometry, topology, and network features. To be more inclusive and complete, we added features that may not be discriminative and may correlate with other features, and relied on subsequent feature selection to ignore such correlative features, if they exist.

SUPPLEMENTAL FIGURE LEGENDS

Supp. Figure S1 Average values of the significant and best ranked features at the ROI level for 80 and 180 nm thresholds.

Supp. Figure S2. The process of generating random blinks for a 3D ROI.

Random graphs are effectively used to filter out noisy blinks of the ROIs extracted from the real cells of our datasets. The random blinks are generated with the same distribution of the blinks in the real ROI. The distribution of the blinks is uniformly distributed in X and Y dimensions and normally distributed in the Z dimension. Also see Figure 3C,D and Figure S3.

Supp. Figure S3. Noisy blinks filtering using significant features at significant thresholds: The effect of changing α to filter out the noisy blinks.

The first column shows all the blinks of two cells from PC3 and PC3-PTRF populations. The nodes are color-coded by the values of the different network measures at two different thresholds. The degree features are significant at 80 nm while the clustering coefficient feature is significant at 180 nm. The second, third, fourth, and the fifth columns shows the results of filtering the noisy blinks at different values of α = 1, 2, 3, 4 respectively. The sixth column shows the histograms of the network measure of the cells compared with the network measures of the random graphs. Also see Figure 3.

SUPPLEMENTAL VIDEO LEGENDS

Supp. Video S1. Animated rotating structures of S1A scaffold (PP4) blob. The blob's predicted molecules, connections, connected components, and the blob's modules at various proximity thresholds are shown from different angles with rotation.

Supp. Video S2. Animated rotating structure of S1B scaffold (PP3) blob. The blob's predicted molecules, connections, connected components, and the blob's modules at various proximity thresholds are shown from different angles with rotation.

Supp. Video S3. Animated rotating structure of S2 scaffold (PP1) blob. The blob's predicted molecules, connections, connected components, and the blob's modules at various proximity thresholds are shown from different angles with rotation. We show the cross-sections and surface reconstruction of the blob.

Supp. Video S4. Animated rotating structure of caveolae (PP2) blob. The blob's predicted molecules, connections, connected components, and the blob's modules at various proximity thresholds are shown from different angles with rotation. We show the cross-sections and surface reconstruction of the blob.

REFERENCES

1 Newman, M. E. J. The structure and function of complex networks. *SIAM review* **45**, 167-256 (2003).

2 Rubinov, M. & Sporns, O. Complex network measures of brain connectivity: uses and interpretations. *Neuroimage* **52**, 1059-1069 (2010).

3 Westin, C. F. Geometrical diffusion measures for MRI from tensor basis analysis. *Proc. ISMRM'97* (1997).

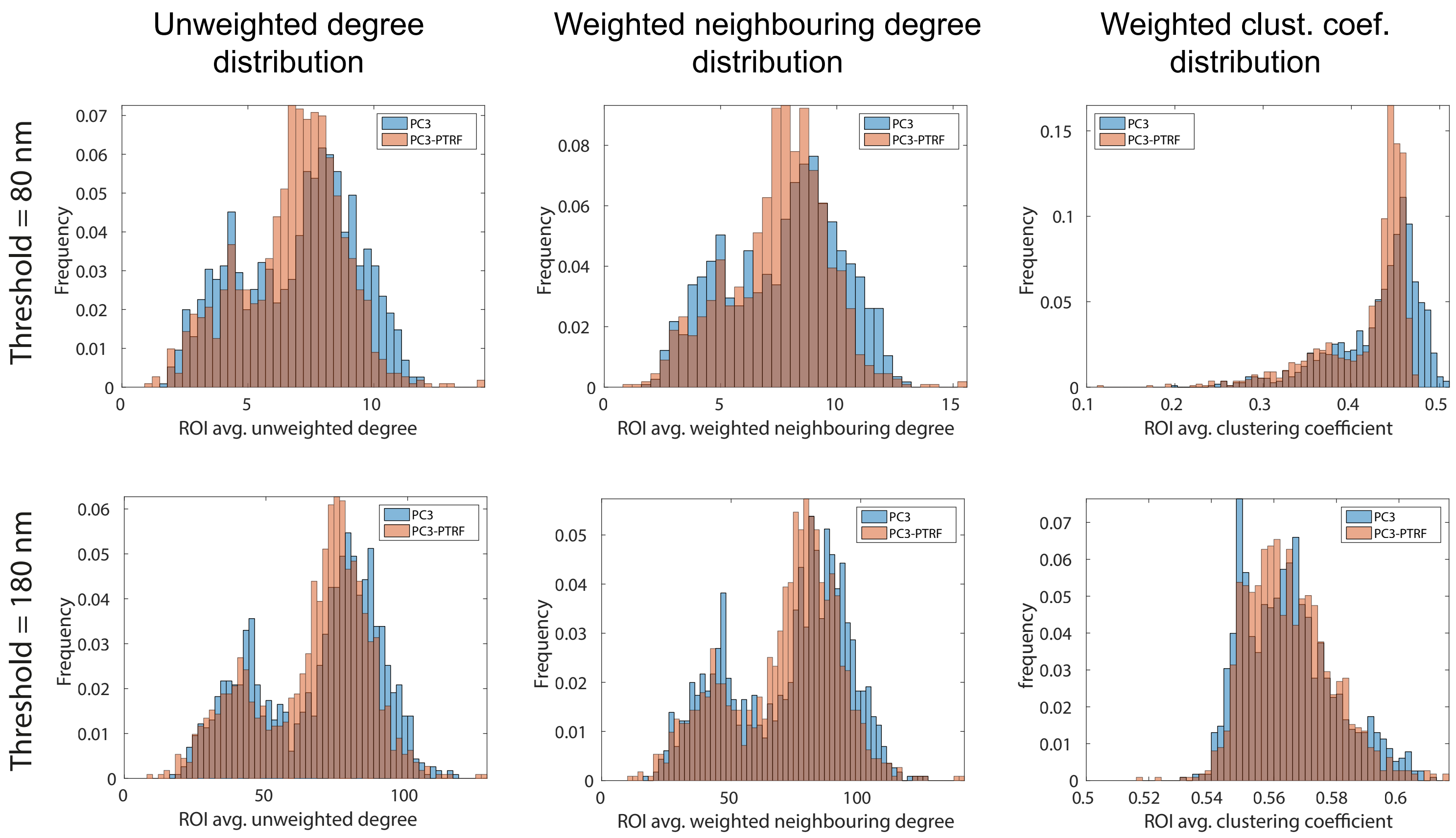

Khater et al, Figure S1

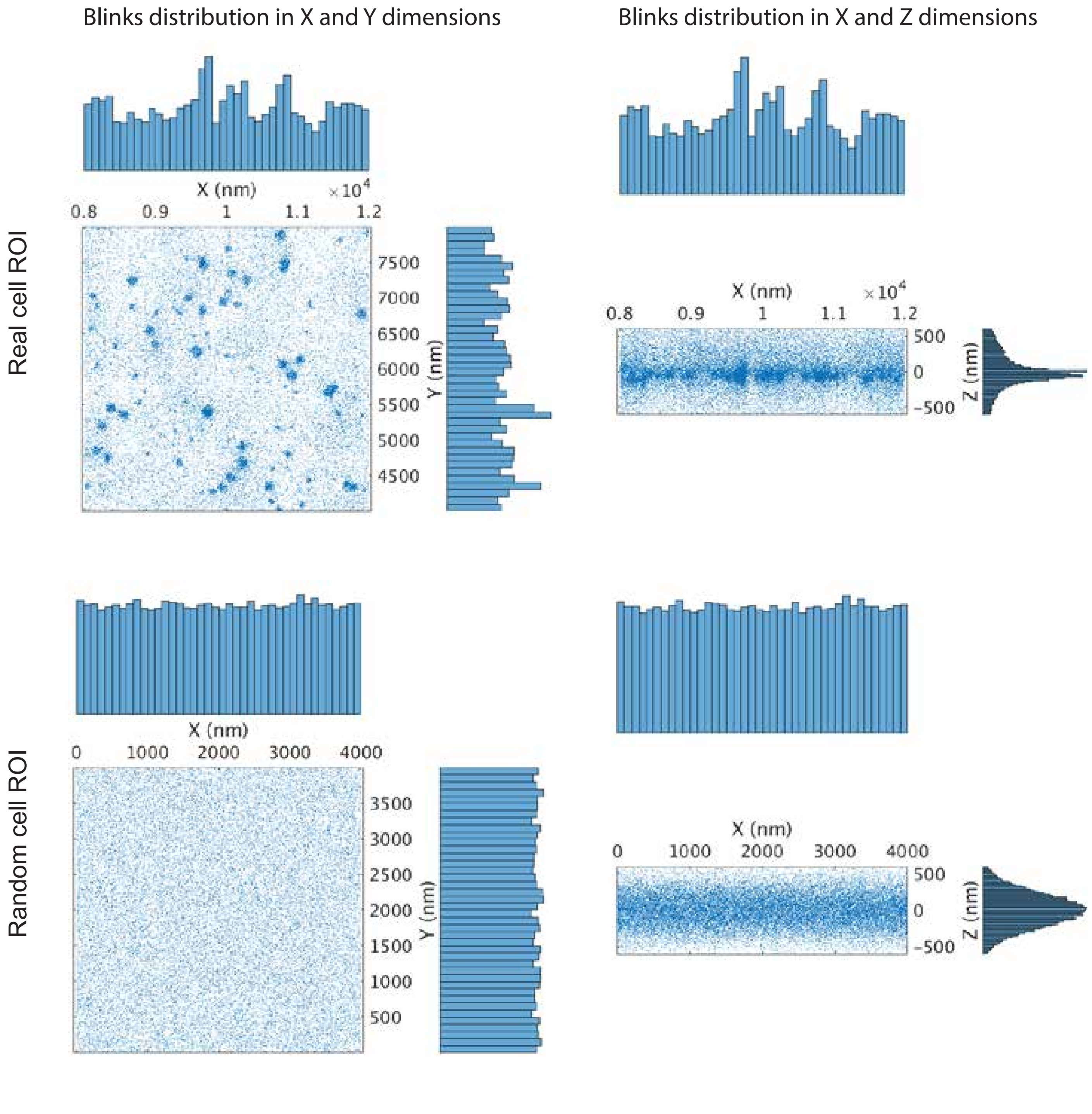

Khater et al, Figure S2

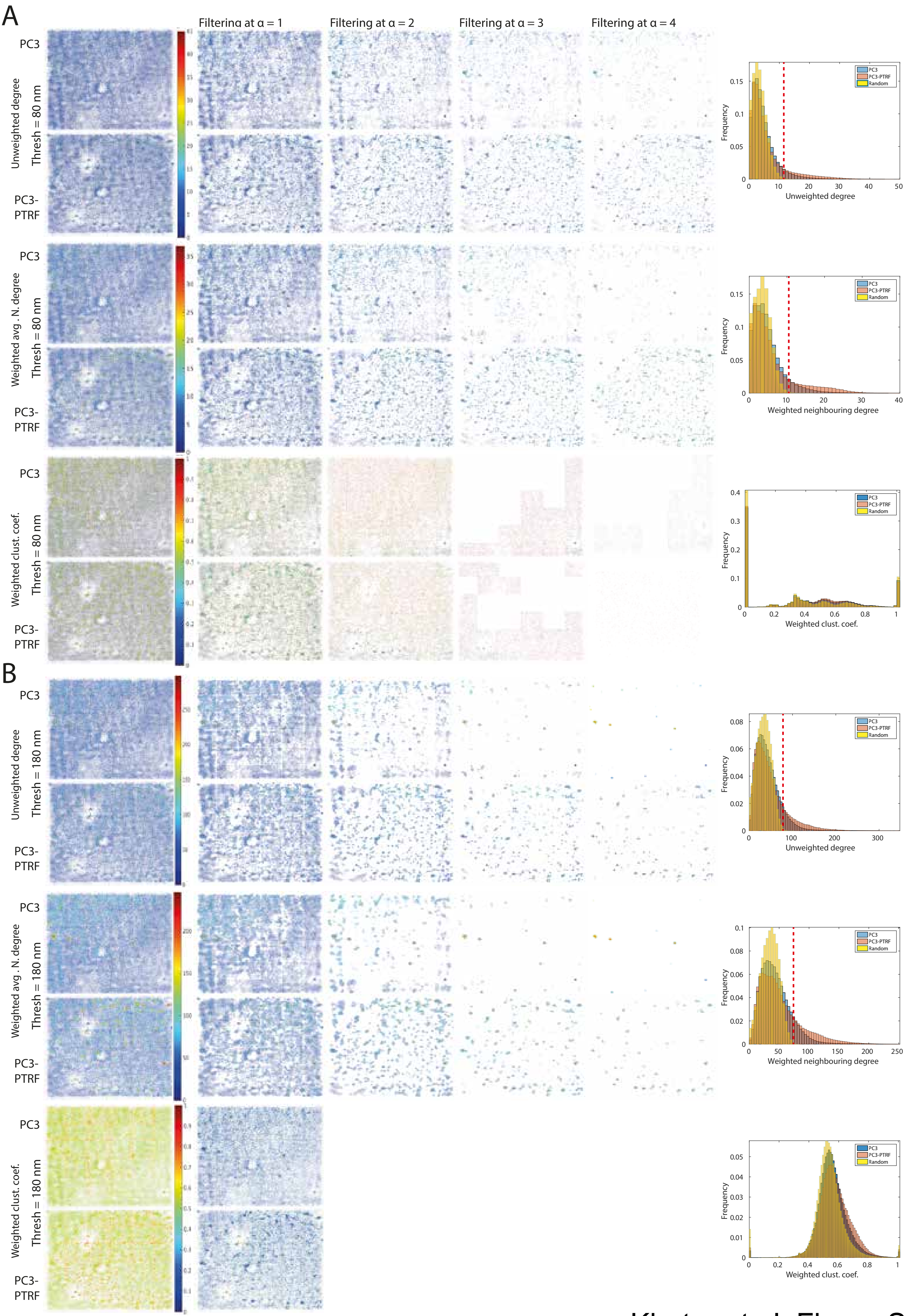

Khater et al, Figure S3

Network Measure	Description
Node level measures	
wAvgDeg	Average weighted degree measure (node strength)
wSTDeg	Standard deviation of the weighted degree
wMedDeg	Median weighted degree
uwAvgDeg	Average unweighted degree measure
uwSTDeg	Standard deviation of the unweighted degree
uwMedDeg	Median unweighted degree
wAvgCC	Average weighted clustering coefficient
wSTCC	Standard deviation of the weighted clustering coefficient
wMedCC	Median weighted clustering coefficient
wAvgNdeg	Average weighted neighbouring degrees
wSTNdeg	Standard deviation of the weighted neighbouring degrees
wMedNdeg	Median weighted neighbouring degrees
uwAvgNdeg	Average unweighted neighbouring degrees
uwSTNdeg	Standard deviation of the unweighted neighbouring degrees
uwMedNdeg	Median unweighted neighbouring degrees
wAvgEcc	Average weighted eccentricity
wSTEcc	Standard deviation of the weighted eccentricity
wMedEcc	Median weighted eccentricity
uwAvgEcc	Average unweighted eccentricity
uwSTEcc	Standard deviation of the unweighted eccentricity
uwMedEcc	Median unweighted eccentricity
Network level measures	
wDen	Weighted network density
wDia	Weighted network diameter
uwDia	Unweighted network diameter
wRad	Weighted network radius
uwRad	Unweighted network radius
wLambda	The average shortest path length (characteristic path) in the weighted network
uwLambda	The average shortest path length (characteristic path) in the unweighted network
numConComp	Number of connected components of the undirected network
largestConComp	Largest connected components in the undirected network
wSmetric	Sum of products of the weighted degrees across all edges
uwSmetric	Sum of products of the unweighted degrees across all edges

Khater et al, Table S1

Feature	Description
Shape features	
FA	Fractional anisotropy
CL	Linear anisotropy
CP	Planar anisotropy
CS	Spherical anisotropy
X-range	Distribution of the point cloud along the X-dimension
Y-range	Distribution of the point cloud along the Y-dimension
Z-range	Distribution of the point cloud along the Z-dimension
Volume	Ellipsoid volume of the 3D point cloud of the blob
Hollowness features	
Avg. Rc	Average distances of the nodes to their centroid
Max. Rc	Maximum distance of the nodes from their centroid
Min. Rc	Minimum distance of the nodes from their centroid
Median Rc	Median distance of the nodes to their centroid
STD Rc	Standard deviation of the nodes distances to their centroid
Network features	
Avg. degree	Average node degree within the blob
Max. degree	Maximum node degree within the blob
Min. degree	Minimum node degree within the blob
Avg. clust. coeff.	Average clustering coefficient value of the nodes
Max. clust. coeff.	Maximum clustering coefficient value of the nodes
Min. clust. coeff.	Minimum clustering coefficient value of the nodes
Transitivity	Transitivity measure of the nodes
Char. path	Characteristic path of the nodes
Avg. eccentricity	Average eccentricity measure
Graph radius	Blob’s network/graph radius
Graph diameter	Blob’s network/graph diameter
Graph density	Blob’s network/graph density
Modularity	Blob’s network/graph modularity measure
Avg. opt. mod.	Average optimized modularity for the blob’s network
# molecules	Number of nodes

Khater et al, Table S2
